# Supplementary material for: Surface Modulation of 3D Porous CoNiP Nanoarrays In Situ Grown on Nickel Foams for Robust Overall Water Splitting
Source: Int J Mol Sci. 2022 May 10;23(10):5290. doi: 10.3390/ijms23105290 (PMC9141634; doi:10.3390/ijms23105290)
Supplement: Supplementary file 1 [file ijms-23-05290-s001.zip › ijms-1680621-supplementary.pdf]

Surface Modulation of 3D Porous CoNiP Nanoarrays In Situ Grown on Nickel Foams for Robust Overall Water Splitting

Jianpeng Li [1,†], Caiyan Gao [2,†], Haiyang Wang [1], Baojun Li [1], Shufang Zhao [3], Young Dok Kim [3], Zhongyi Liu [1], Xin Du [1,*] and Zhikun Peng [1]

[1] College of Chemistry, Research Center of Green Catalysis, Henan Institute of Advance Technology,
Zhengzhou University, Zhengzhou 450001, China; lijianpeng@gs.zzu.edu.cn (J.L.);
18037897076@163.com (H.W.); lbjfcl@zzu.edu.cn (B.L.);
zhongyiliu@zzu.edu.cn (Z.L.); pengzhikun@zzu.edu.cn (Z.P.)

[2] School of Resources and Environmental Engineering, Wuhan University of Technology,
Wuhan 430070, China; 17803897978@163.com

[3] Department of Chemistry, Sungkyunkwan University, Suwon 16419, Korea;
taylorbjt096-@naver.com (S.Z.); ydkim91@skku.edu (Y.D.K.)

* Correspondence: duxin_1911@163.com (X.D.)

† These authors contributed equally to this work..

Density Functional Theory Simulation

The first-principles density functional theory (DFT) calculations was performed via Vienna abinitio simulation package (VASP) from the website (https://www.materialsproject.org/materials/mp-990448/#),

In order to obtain the accurate electronic structures and free energy, The calculations were performed using the hybrid functional as proposed by PBE interactions are represented using the projector augmented wave (PAW) potential, and the Kohn-Sham one-electron valence states were expanded on the basis of plane waves with a cutoff energy of 400 eV. The Hellmann-Feynman forces convergence criterion was set as less than 0.05 eV Å^{-1}, The K-point of 3×3×1 was used for the optimization of the surface of Co_2P, Ni_2P and CoNiP (001) and the atomic layer with the thicknesses about 3.0-15.0 Å is anchored.

The zero point energy (ZPE) correction was performed referring to the approaches previously reported. In the DFT process, we calculated the Gibbs free energy according to the equations as follow:

$$G^0 = E_{DFT} + ZPE - TS^0$$

Where G^0 is the Gibbs free energy, E_{DFT} is total free energy, ZPE is the vibration energy; TS^0 is the entropy change (T = 298.15 K).

The OER was contained of four-electron transfer half-reaction in electrocatalytic water splitting, thereby, the total free energy was obtained from the following formulas:

$$\Delta G = \Delta G_1 + \Delta G_2 + \Delta G_3 + \Delta G_4$$

$$\Delta G_1 : OH^- + * \rightarrow OH* + e^-$$

$$\Delta G_2 : OH^- + OH^* \rightarrow O^* + H_2O + e^-$$

$$\Delta G_3 : OH^- + O^* \rightarrow OOH^* + e^-$$

$$\Delta G_4 : OH^- + OOH^* \rightarrow O_2 + * + H2O + e^-$$

Where ΔG is the total free energy; ΔG_1 is the absorption energy of first electron tranfer reaction; ΔG_2 is the absorption energy of second electron tranfer reaction; ΔG_3 is the absorption energy of third electron tranfer reaction; ΔG_4 is the absorption energy of fourth electron tranfer reaction.

In addition, the adsorption energies of intermediates (*OH and *OOH) were calculated by the following formulas:

$$\eta^{OER} = (G^{OER} / e) - 1.23V$$

Where η^{OER} is the overpotential, G^{OER} is the max value among (ΔG_1 , ΔG_2 , ΔG_3, ΔG_4).

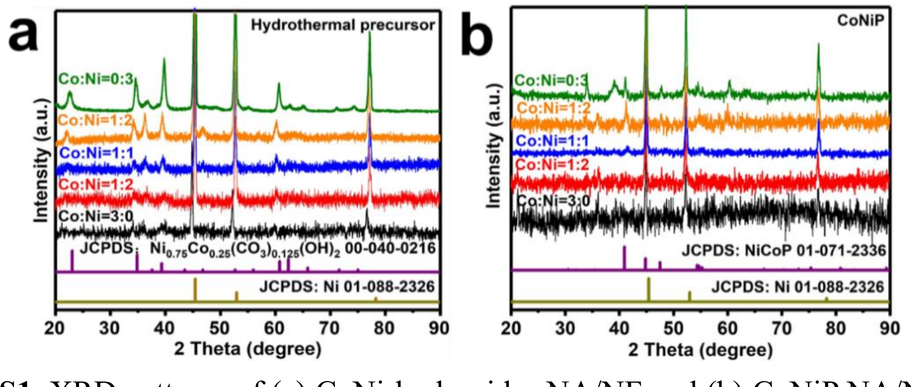

Figure S1. XRD patterns of (a) CoNi-hydroxides NA/NF and (b) CoNiP NA/NF with different Co/Ni ratios.

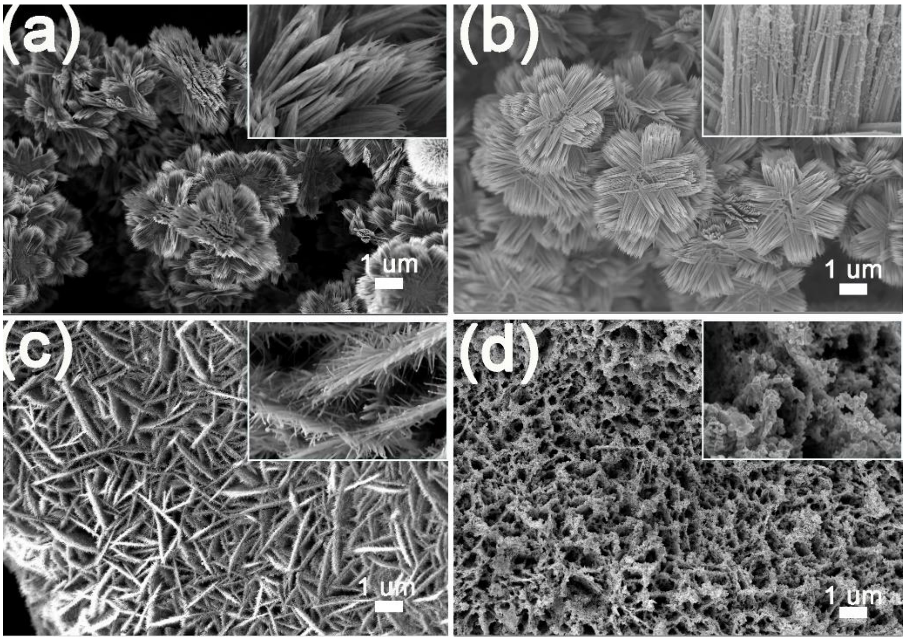

Figure S2. SEM images of (a) Co_2P NA/NF, (b) CoNiP NA/NF-1, (c) CoNiP NA/NF-3 and (d) Ni_2P NA/NF

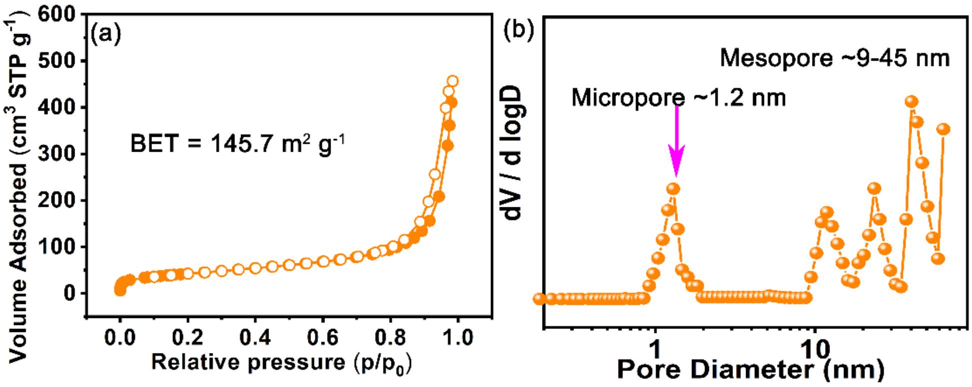

Figure S3 (a) Nitrogen adsorption-desorption isotherms and (b) corresponding pore size distribution of CoNiP NA/NF-2

Table S1. The comparison of HER performance of the samples in 1 M KOH solution.

Catalysts	Current density (mA/cm^2)	Overpotential	Ref
CoP-MNA/NF	10	189	
Ni	10	400	ACS Catal. 2013, 3, 166
CoP/CC	10	209	J. Am. Chem. Soc. 2014, 136, 7587
Co_2P nanorods	10	171	Nano Energy 2014, 9, 373
Fe-CoP	10	190	Adv. Sci., 2018, 5, 1800949.
CoFePBA@CoP	10	171	Small Methods, 2021, 5.
CoMnP/Ni_2P	10	209	J. Mater. Chem. A, 2021, 9,22129-22139.

Ni_2P-Fe_2P	10	261	J. Mater. Chem. A, 2021, 9, 22129-22139.
CoNiP NA/NF	10	162	**This works**

Table S2. Comparison of cell potentials at a current density of 10 mA cm^{-2} for TMPs-based bifunctional electrocatalysts toward overall water splitting

**Catalysts**	**Cell potential (V)**	**Ref**
Ni_5P_4 film	1.7	Angew. Chem. Int. Ed. 2015, 54, 12361–12365
CoNiP/rGO	1.59	Adv. Funct. Mater. 2016, 26, 6785 6796
CoNiP	1.57	Adv. Funct. Mater. 2016, 26, 7644 7651
NiFeP	1.65	Adv. Energy Mater. 2017, 7, 1700107
CoNiP	1.64	Adv. Mater. Interfaces 2016, 3, 1500454
NiFeP	1.67	ACS Appl. Mater. Interfaces 2017, 9, 26134 26142
$Ni_{1.5}Fe_{0.5}P$	1.59	Nano Energy 2017, 34, 472 480
$Ni_{1-x}Co_xP$	1.59	Nanoscale 2016, 8, 19129 19138
CoNiP NA/NF	1.613	**This works**

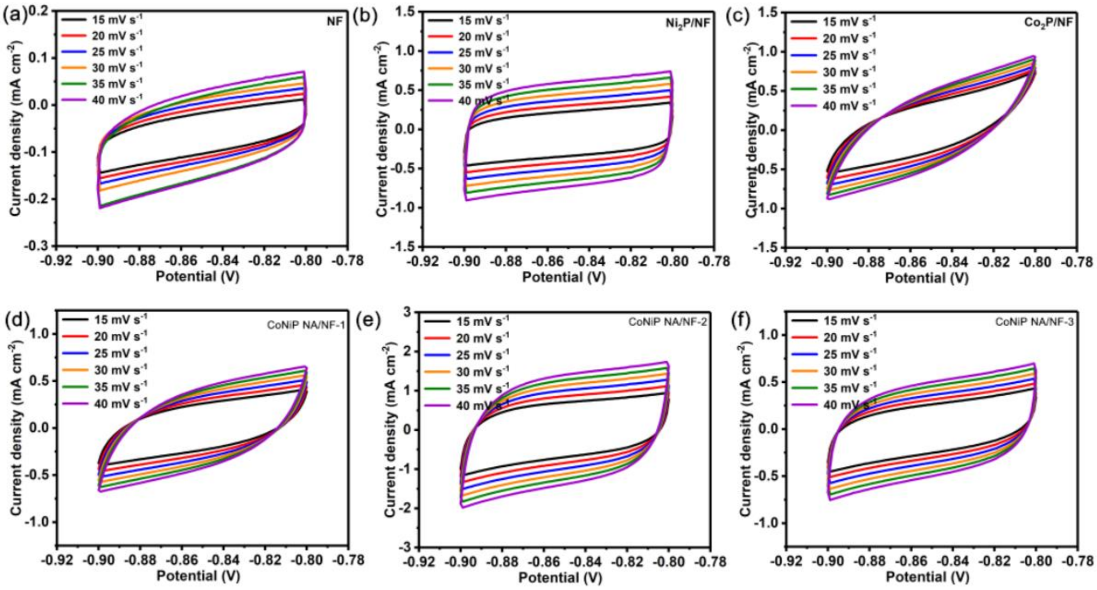

Figure S4. (a-f) CV curves measured for HER with scan rate from 15 to 40 mV s^{-1} for various samples.

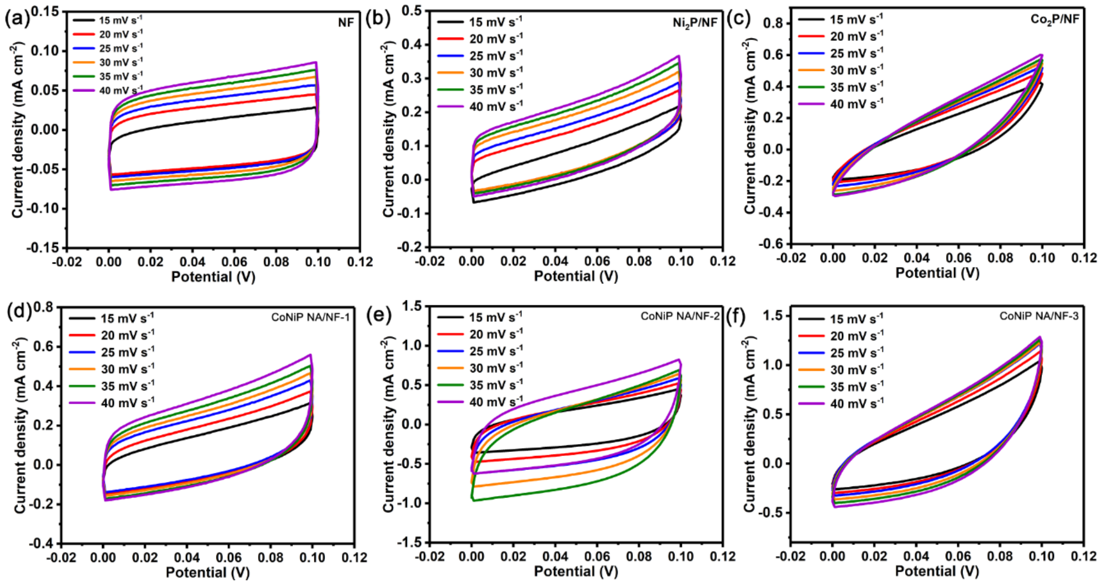

Figure S5. (a-f) CV curves measured for OER with scan rate from 15 to 40 mV s^{-1} for various samples.

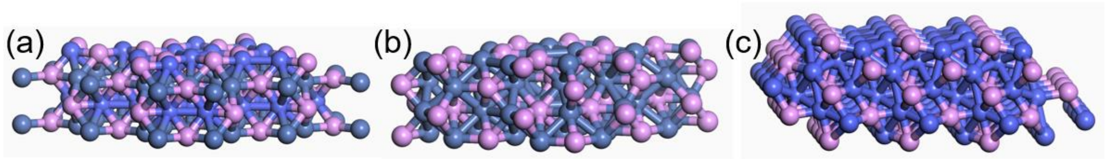

Figure S6 Crystal structure model. (a) CoNiP, (b) Ni_2P, and (c) Co_2P.

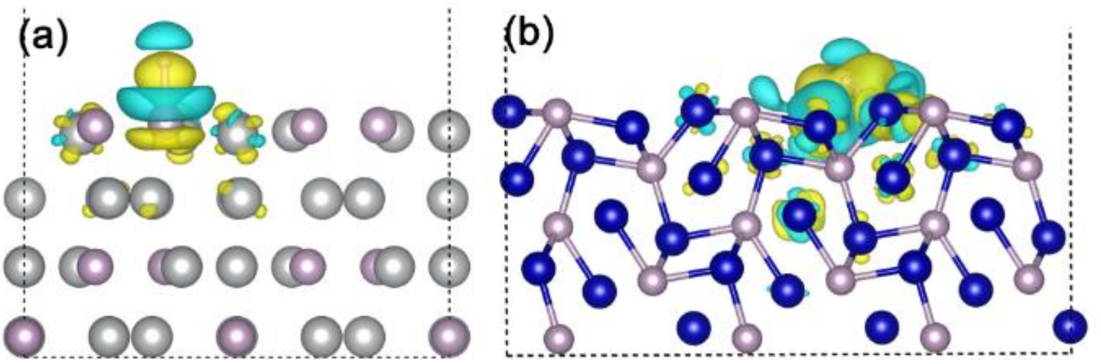

Figure S7 Charge density difference plots at the surface of CoNiP (a) Ni_2P, (b)Co_2P for HER.

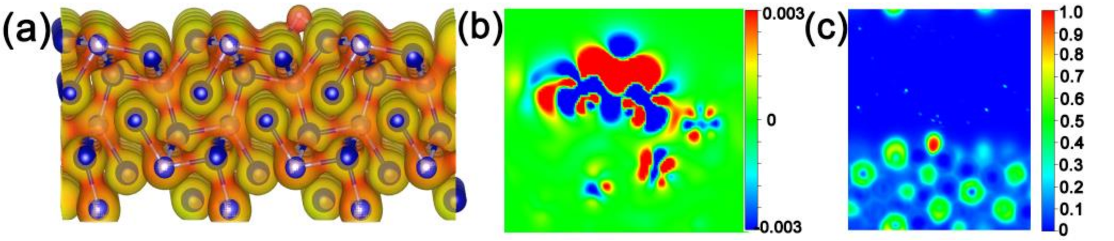

Figure S8 Electrostatic potential distribution after adsorption of H* on the (a) Co_2P surface. The corresponding cross section of charge density difference of (b) Co_2P. The corresponding electron localization function (ELF) of (c) Co_2P.

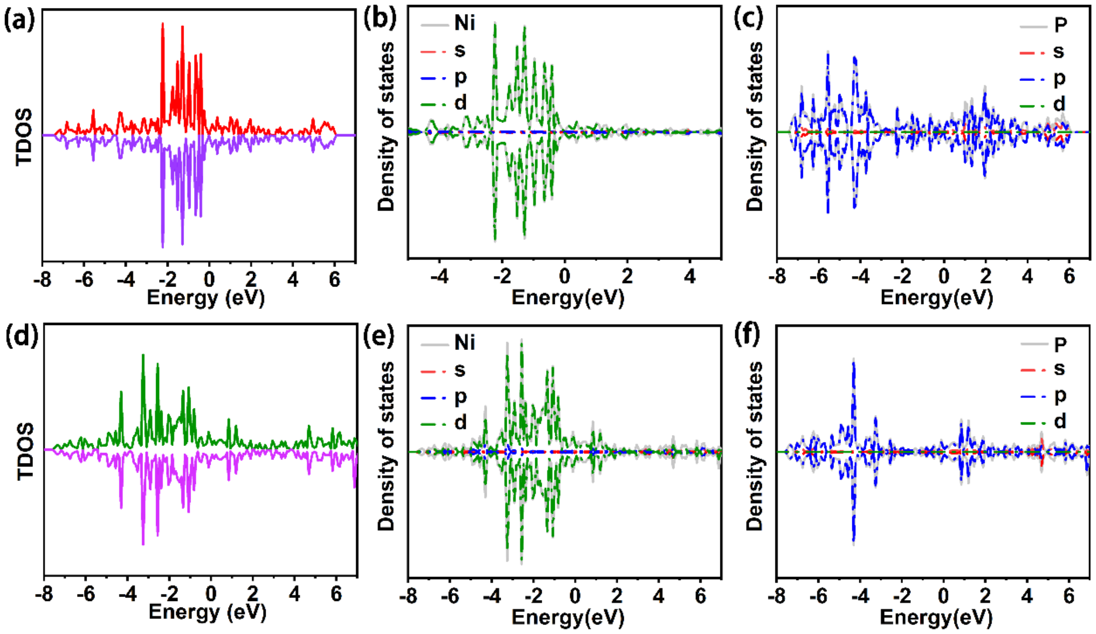

Figure S9 Calculated total and partial electronic density of states for (a-c) CoNiP, and (d-e) Ni_2P.
